# Amplified P2X_3_ pathway activity in muscle afferent dorsal root ganglion neurons and exercise pressor reflex regulation in hindlimb ischaemia–reperfusion

**DOI:** 10.1113/EP091616

**Published:** 2024-01-11

**Authors:** Lu Qin, Qin Li, Jianhua Li

**Affiliations:** ^1^ Heart and Vascular Institute Penn State College of Medicine Hershey Pennsylvania USA

**Keywords:** blood pressure, dorsal root ganglion, ischaemia–reperfusion, peripheral artery disease, purinergic P2X_3_

## Abstract

Hindlimb ischaemia–reperfusion (IR) is among the most prominent pathophysiological conditions observed in peripheral artery disease (PAD). An exaggerated arterial blood pressure (BP) response during exercise is associated with an elevated risk of cardiovascular events in individuals with PAD. However, the precise mechanisms leading to this exaggerated BP response are poorly elucidated. The P2X_3_ signalling pathway, which plays a key role in modifying the exercise pressor reflex (EPR), is the focus of the present study. We determined the regulatory role of P2X_3_ on the EPR in a rat model of hindlimb IR. In vivo and in vitro approaches were used to determine the expression and functions of P2X_3_ in muscle afferent nerves and EPR in IR rats. We found that in IR rats there was (1) upregulation of P2X_3_ protein expression in the L4–6 dorsal root ganglia (DRG); (2) amplified P2X currents in isolated isolectin B4 (IB4)‐positive muscle DRG neurons; and (3) amplification of the P2X‐mediated BP response. We further verified that both A‐317491 and siRNA knockdown of P2X_3_ significantly decreased the activity of P2X currents in isolated muscle DRG neurons. Moreover, inhibition of muscle afferents’ P2X_3_ receptor using A‐317491 was observed to alleviate the exaggerated BP response induced by static muscle contraction and P2X‐induced BP response by α,β‐methylene ATP injection. P2X_3_ signalling pathway activity is amplified in muscle afferent DRG neurons in regulating the EPR following hindlimb IR.

## Abstract (highlights)

- **What is the central question of this study?**
  Is the P2X_3_ signal pathway engaged in the exaggerated exercise pressor reflex (EPR) response in the ischaemia–reperfusion (IR) injury of peripheral artery disease (PAD)?
- **What is the main finding and its importance?**
  IR upregulates expression of the P2X_3_ receptor and increases the P2X currents in muscle afferent DRG neurons. Pharmacological inhibition using A‐317491 (P2X_3_ antagonist) and the genetic knockdown of P2X_3_ reduce the P2X currents in the muscle afferent DRG neurons. A‐317491 also attenuates the exacerbated EPR response following IR.
  The findings provide evidence that interventions attenuating the P2X_3_ signalling pathway may enhance tolerance of and adherence to exercise rehabilitation for PAD patients.

## 1 INTRODUCTION

With the stimulus of skeletal muscle movement during exercise, activation of the sympathetic nervous system increases peripheral vasoconstriction and subsequently increases arterial blood pressure (BP), heart rate (HR) and myocardial contractility (Sinoway et al., 1989; Victor et al., 1988). Two major neural regulation mechanisms are considered to be involved in this process: central command (Goodwin et al., 1972; Waldrop et al., 1996) and the exercise pressor reflex (EPR) (McCloskey & Mitchell, 1972; Mitchell et al., 1983). Central command suggests that motor and sympathetic activation occur in parallel, that is, there is a volitional signal emanating from central motor areas leading to increased sympathetic nervous activity (SNA) during exercise. This system is linked to skeletal muscle metabolic needs via parallel brain activation of motor and autonomic centres. The EPR is mediated by group III and IV afferents arising from contracting skeletal muscle in response to mechanical deformation and metabolic stimulation (Kaufman et al., 1984).

The EPR is exaggerated in peripheral artery disease (PAD) (Baccelli et al., 1999; Ritti‐Dias et al., 2011). In PAD patients this reflex is a dominant determinant as exercise‐induced BP rise (i.e., during walking) in PAD patients is greater than that seen in healthy control subjects. Moreover, during static exercise the BP response for the affected limb is greater than that for the unaffected limb (Lorentsen, 1972). Notably, the augmented BP response during exercise is associated with a higher incidence and risk of cardiovascular diseases (Lewis et al., 2008) and there is a lower survival rate in PAD patients than in asymptomatic normotensive subjects (de Liefde et al., 2008; Weiss et al., 2010). Nonetheless, the molecular mediators and underlying mechanisms alleviating the exaggerated exercise‐induced BP response in PAD need to be revealed.

Ischaemia–reperfusion (IR) injury is a main pathophysiological condition of various cardiovascular diseases, including myocardial infarction, stroke and PAD (Cryer, 1997; Maxwell & Lip, 1997). During exercise and/or moderate ischaemia, ATP is increased in skeletal muscle (Hellsten et al., 1998; Li et al., 2003; Mo & Ballard, 2001), and muscle afferents play a role in regulating the EPR. Additionally, injecting α,β‐methylene ATP (α,β‐meATP, a receptor agonist of P2X) into the arterial blood supply of hindlimb muscles to stimulate P2X of muscle afferent nerves increases SNA and BP to a greater degree in PAD rats with occluded femoral artery (Liu et al., 2011). Notably, P2X_3_ expression is upregulated in dorsal root ganglion (DRG) neurons innervating the hindlimb muscles with the occluded femoral artery (Liu et al., 2011). P2X_3_ likely plays a key role in regulating sympathetic and cardiovascular responses to stimulation of metabolically sensitive muscle afferent nerves. Nevertheless, the precise mechanisms by which P2X_3_ is engaged in the exaggerated SNA and BP responses in the IR injury of PAD, a major disease condition, are still poorly understood.

For those reasons, in this report, we used a rat model of IR with 6 h ischaemia followed by 18 h reperfusion (IR rats) to determine the role played by P2X_3_ in regulating the EPR in PAD rats. Specifically, western blot analysis was used to examine the levels of P2X_3_ in DRG of sham rats and IR rats. Patch clamp experiments were used to determine the activities of P2X current in muscle DRG neurons, and a whole animal preparation was used to determine if the BP response to activation of P2X_3_ and static muscle contraction was attenuated after blocking P2X_3_ receptors in muscle afferent nerves. We hypothesized that the expression and activity of the P2X_3_ signalling pathways in thin‐fibre muscle afferent nerves are increased in IR rats, and a P2X_3_ receptor antagonist, A‐317491, by intra‐arterial administration into the hindlimb muscles, will attenuate an exaggerated BP response to stimulation of P2X as well as to static muscle contraction.

## 2 METHODS

### 2.1 Ethical approval

All experimental procedures were approved by the Institutional Animal Care and Use Committee of Penn State College of Medicine (Protocol no.: PRAMS201147671) and were conducted in accordance with the National Institutes of Health *Guide for the Care and Use of Laboratory Animals*. Male Sprague–Dawley rats (4−6 weeks old) were housed in accredited temperature‐ and ventilation‐controlled facilities with a 12:12 h light–dark cycle and ad libitum access to standard rat chow and water.

### 2.2 Surgeries for hindlimb IR

To perform the surgery of the hindlimb IR, rats were anaesthetized by inhalation of 2–5% isoflurane in 100% oxygen. Buprenorphine hydrochloride (0.05 mg/kg, subcutaneously) was administered prior to surgery for post‐operative pain relief. A ligature was placed tightly with a slipknot around the femoral artery ∼3 mm distal to the inguinal ligament. After the femoral artery occlusion, the rats were removed from anaesthesia and kept in the surgical room for 6 h. Then anaesthesia was introduced again and the surgery site was re‐opened. Blood flow reperfusion was induced by releasing the slipknot to return blood flow into the femoral artery. Surgical forceps were used to gently massage the artery to assist the removal of the blood clot and the recovery of blood flow. After observing the blood flow entering the previously ligated femoral artery, the surgical wound was carefully closed. To obtain sham‐controls, the same procedures were followed except that a suture was placed below the artery but the artery remained intact. Following the surgery, the animals were kept in the surgical room for 2–3 h for observation, and then returned to the animal facility. All the In vivo and in vitro experiments were performed 18 h after the end of the blood flow reperfusion.

### 2.3 Western blotting analysis

As described previously (Qin et al., 2020), the total protein of rat L4–L6 DRG tissue in sham limbs (*n* = 9) and IR limbs (*n* = 8) was extracted. Thirty micrograms of protein was loaded in 10% Mini‐Protean TGX Precast gels (Bio‐Rad Laboratories, Hercules, CA, USA) after being boiled at 95°C for 5 min in SDS sample buffer, then electrophoretically transferred to polyvinylidene difluoride membrane. After blocking with 5% non‐fat milk in 0.1% Tween–Tris‐buffered saline (TBST) for 1 h, the membrane was incubated with rabbit anti‐P2X_3_ (1:1000, Alomone Laboratories, Jerusalem, Israel) primary antibody at 4°C overnight and then incubated with horseradish peroxidase‐conjugated anti‐rabbit secondary antibody (1:2000, Cell Signaling Technology, Danvers, MA, USA) at room temperature for 1 h. Immunoreactivity was visualized using an enhanced chemiluminescence system (Cell Signaling Technology). The membrane was stripped and incubated with anti‐β‐actin primary antibody (1:1000, Sigma‐Aldrich, St Louis, MO, USA) as the internal protein expression control. The optical densities of targeted bands were analysed using the NIH (Bethesda, MD, USA) ImageJ software.

### 2.4 siRNA‐knockdown in DRGs

A siRNA oligo duplex of rat P2X_3_ was designed to target a unique gene sequence for P2X_3_: rat P2X_3_ siRNA (5′‐CCGUCCAGCUGCUGAUUAUTT and 5′‐AUAAUCAGCAGCUGGACGGTT). All siRNA oligo duplex and the negative control siRNA were obtained from Sigma‐Aldrich.

As described previously (Hassan et al., 2014; Mahmoud et al., 2012), L4–5 DRGs were dissected, cut with three to four slides, and incubated in cold Hanks’ balanced salt solution before transfection. The DRGs were firstly electroporated using the Neon Electroporation System (Thermo Fisher Scientific, Waltham, MA, USA) with a protocol of 1000 V pulses/20 ms/three times in a 100 μL electroporation transfection system, containing T solution, 3 μL 100 μM siRNA oligos, and 3 μL 100 mM 2,3‐butanedione monoxime (BDM) (Abcam, Waltham, MA, USA). Then, the DRGs were transferred onto a 22‐mm dish containing 1 mL transfection mixture in Opti‐MEM medium (20 μL 100 μM siRNA oligos, 20 μL BDM, 10 μL Lipofectamine RNAiMAX, Thermo Fisher Scientific). After this, the DRGs were cultured in a CO_2_ incubator for 4 h. The transfection mixture was removed and changed into final Dulbecco's modified Eagle's medium to be cultured for 2 days. Another transfection was applied for 2 days before the patch clamp experiments were performed.

### 2.5 Electrophysiology

#### 2.5.1 Labelling of hindlimb muscle afferent DRG neurons

Briefly as described (Li et al., 2021, 2020), 3 days before IR models were constructed, following anaesthesia, an incision in the calf area of one limb was made and the gastrocnemius muscle was exposed. The lipophilic dye 1,1′‐dioctadecyl‐3,3,3′,3′‐tetramethylindocarbocyanine perchlorate (DiI, 60 mg/mL) was injected into the white portion of the gastrocnemius muscle. A total volume of 1 μL DiI tracer was administered at different locations, with the needle left in the muscle for 1 min to prevent tracer leakage. The same procedure was carried out in the contralateral limb. After that, the rats were returned to their cages for fluorescent DiI retrograde transport to the DRGs to label muscle DRG neurons.

#### 2.5.2 Patch clamp recording

All recording was performed on DRG neurons within 6 h after their being dissociated. Immediately before recording, neurons were incubated with isolectin B4 (IB4)–Alexa Fluor 488 (3 μg/ml in external solution; Thermo Fisher Scientific) for 5 min and then rinsed with external solution for another 5 min. Two distinct subpopulations of muscle DRG neurons, namely, IB4‐positive and IB4‐negative, were identified under a microscope. DRG neurons were first visualized using differential interference contrast (DIC; ×20–40) optics and then an IB4‐positive neuron was visualized (by its green colour) using a combination of fluorescence illumination and DIC optics on a Nikon TE2000 inverted microscope. Meanwhile, those DRG neurons were also identified as Dil‐positive (red color) under an inverted microscope with a fluorescence filter, and images were displayed on a video monitor. IB4‐positive muscle DRG neurons were first identified. IB4‐positive and IB4‐negative muscle DRG neurons were randomly numbered from 20 version fields in five coverslips under a microscope.

The extracellular solutions contained (in mM): 140 NaCl, 5 KCl, 2 CaCl_2_, 1 MgCl_2_, 10 MES, 10 glucose, pH adjusted to 7.4 or the values indicated with 1 M NaOH. Holding at −70 mV, the seals (2–8 GΩ) were obtained with 3–5 MΩ resistance with glass electrodes filled with internal solution (in mM): 140 KCl, 2 MgCl_2_, 5 EGTA, 10 HEPES, 0.3 NaGTP, 2 MgATP, pH adjusted to 7.3 with KOH. The whole‐cell configuration was applied, and the resistance was 70–90% compensated to under 10 MΩ if necessary. In voltage‐clamp mode, a gap‐free protocol of 30 s was used to record P2X currents.

P2X currents of muscle DRG neurons (cell diameters ≤35 μm) were recorded in the whole‐cell configuration using a MultiClamp 700B amplifier supplied with Digitizer 1440A (Molecular Devices, San Jose, CA, USA). Signals were acquired with pClamp 10.1 and analysed with pClampfit 10.7 software (Molecular Devices). All experiments were performed at 20–22°C.

All the chemicals stored in the stock solutions were diluted in extracellular solution immediately before being used and individually held in a series of independent syringes of the pressurized VC3‐8MP perfusion system with an ID#200 μm outlet tip (ALA Scientific Instruments, Inc., Farmingdale, NY, USA). Specifically, 30 μM α,β‐meATP dissolved in saline was used to evoke the P2X currents. Ten micromolar A‐317491 (MedChemExpress, Monmouth Junction, NJ, USA) was used to attenuate P2X_3_. To prepare 10 μM of A‐317491 solution, 5 mg of A‐317491 was first dissolved in 100 μL of dimethyl sulfoxide (DMSO) as a stock solution and then diluted to 10 μM as applied. The distance from the outlet tip mouth to the neuron examined was within 100 μm. The DRG neurons in the recording chamber were continuously bathed in extracellular solution of pH 7.4.

### 2.6 Examination of the BP response

The rats were anaesthetized by inhalation and ventilated. The jugular vein and common carotid artery were cannulated for fluid delivery and a pressure transducer connected to monitor arterial BP. HR was calculated by beat to beat from the arterial pressure pulse. A catheter (PE10) was inserted into the femoral artery for drug delivery. To induce the P2X‐mediated EPR response, the P2X agonist α,β‐meATP (0.125 mM, Sigma‐Aldrich) dissolved in saline (0.1 mL) was given via the femoral artery; and to block the P2X‐mediated EPR response, the P2X antagonist A314791 (10 mg/kg, MedChemExpress) dissolved in 0.2 mL DMSO (vehicle control) was given intra‐arterially according to the rat's body weight. The duration of the injection of α,β‐meATP was 1 min and the duration of the injection of DMSO and A‐317491 was 10 min using an injection pump (Model CMA/102, CMA/Microdialysis AB, Harvard Apparatus, Holliston, MA, USA). During the experiments, baseline BP and fluid balance were maintained with a continuous infusion of saline, and body temperature was maintained at ∼37°C with a heating pad.

Decerebration was performed to eliminate the effects of anaesthesia on the reflex pressor response (Smith et al., 2001). Prior to the procedure, dexamethasone (0.2 mg, i.v.) was injected to minimize brainstem oedema. A transverse section was made anterior to the superior colliculus and extending ventrally to the mammillary bodies, and then all tissues from the rostral to the section were removed (Smith et al., 2001). Following this procedure, the anaesthesia was withdrawn and a ventilator was applied to the rats, and 60 min was allowed before the experiment began.

In brief, a laminectomy procedure was performed to expose the lower lumbar and upper sacral portions of the spinal cord, and the peripheral ends of the transected L4 and L5 ventral roots were placed on platinum bipolar stimulating electrodes. Static muscle contractions were induced by electrical stimulation of the L4 and L5 ventral roots (30 s, 3× motor threshold with a duration of 0.1 ms at 40 Hz). The reflex BP and HR responses during muscle contraction and arterial injection of α,β‐meATP were examined in sham rats and IR rats before the injection of DMSO and A‐317491, and immediately after completing their injection.

### 2.7 Statistical analysis

Unless specified, the data in this study are presented as the mean ± standard deviation (SD). SPSS for Windows version 29.0 (IBM Corp., Armonk, NY, USA) was utilized for all statistical analyses. Mixed ANOVA was applied to compare the differences in BP responses between the groups (A‐317491 and vehicle control) and time point (before and immediately after the drug administration). One‐way ANOVA was used to compare the P2X currents before, immediately after the administration of A‐317491, and after the wash‐out period. As appropriate, Bonferroni's *post hoc* test was applied to compare the differences between specific groups. An independent Student's *t*‐test was applied to compare the differences in P2X_3_ protein expression and currents between groups. The chi‐square test was applied to compare the difference in the percentage of neurons with P2X currents between groups. A *P*‐value less than 0.05 was considered as statistically significance.

## 3 RESULTS

### 3.1 Expression of P2X_3_ in L4–6 DRGs

**FIGURE 1:**
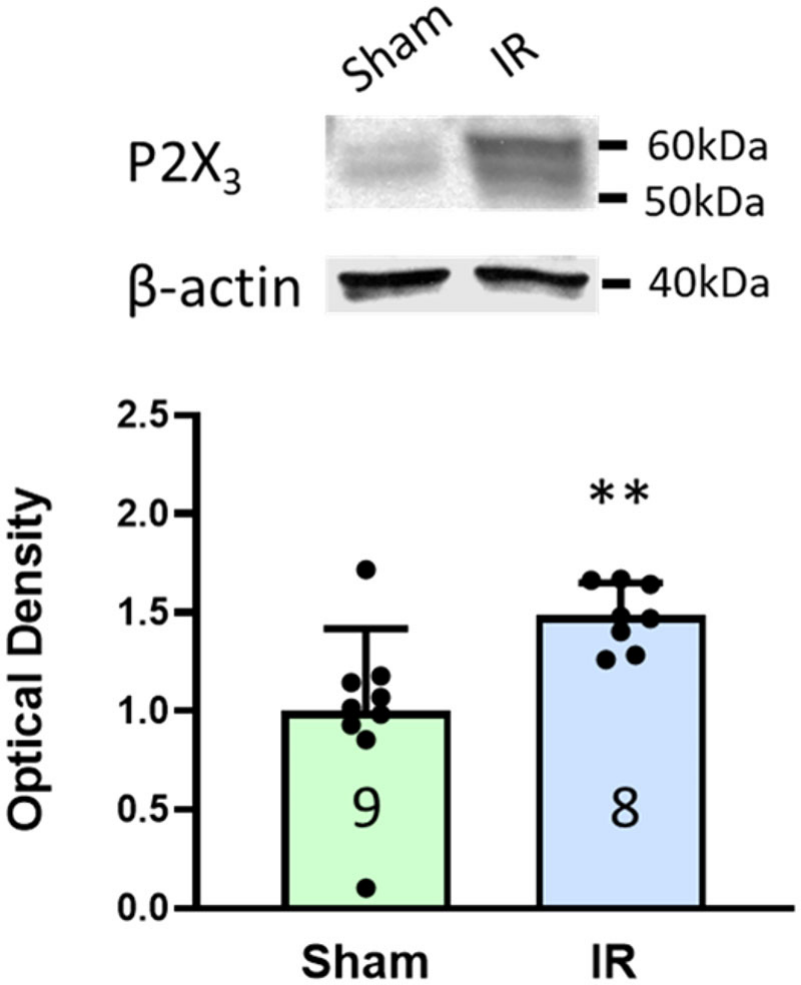
Expression of P2X_3_ in L4–6 DRGs. Western blot analysis showing that, compared with sham rats, there was a significant increase in P2X_3_ protein expression in L4–6 DRGs of IR rats. Data are presented as means ± SD. Numbers in parentheses represent the sample size of each group. **P < 0.01 between IR rats and sham control rats.

Western blotting showed that, compared with sham rats, there was a significant increase in P2X_3_ protein expression in L4–6 DRGs of IR rats (P2X_3_: 1.48 ± 0.16 in IR rats, *n* = 8 vs. 1.00 ± 0.41 in sham rats, *n* = 9; *P* < 0.01; Figure 1).

### 3.2 Contribution of P2X_3_ to current activities of muscle DRG neurons evoked by α,β‐meATP

**FIGURE 2:**
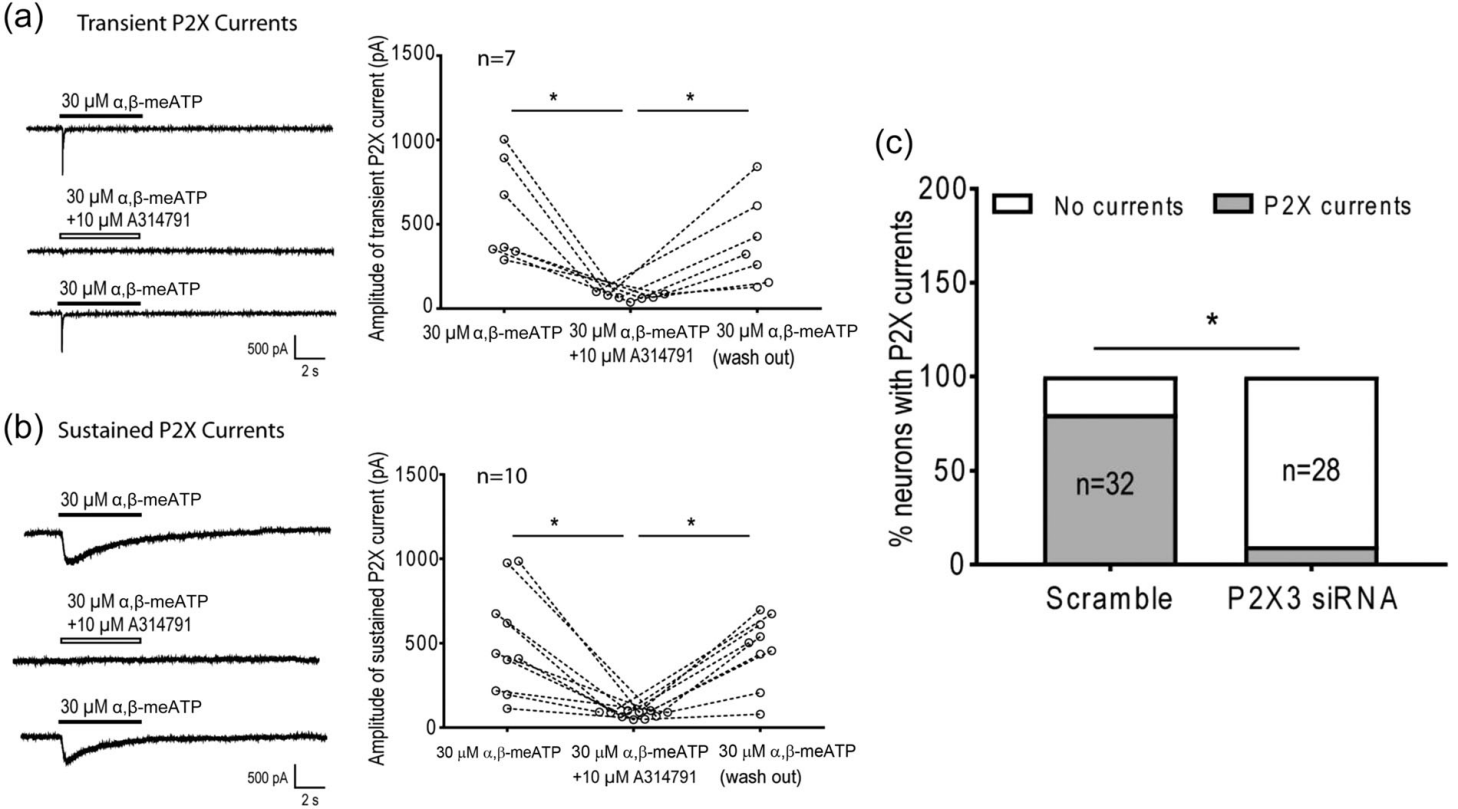
Effect of A‐314791 and siRNA knockdown on P2X_3_ currents in muscle DRG neurons. Data presented as means ± SD. Numbers in parentheses represent the number of neurons with P2X currents detected. (a) The transient P2X current in the muscle DRG neuron was significantly reduced by A‐317491 (*P* < 0.05). The decreased transient P2X currents was recovered after the wash‐out period (*P* < 0.05). *Between groups, *P* < 0.05. (b) Sustained P2X current in the muscle DRG neuron was significantly reduced by A‐317491 (*P* < 0.05). The decreased sustained P2X current was recovered after the wash‐out period (*P* < 0.05). *Between groups as indicated, *P* < 0.05. (c) Compared with the scramble control, the number and percentage of DRG neurons with P2X currents detected were significantly reduced in muscle afferent DRG neurons in P2X3 siRNA knockdown group (*P* < 0.05). *Between scramble group and siRNA knockdown group, *P* < 0.05.

We first determined if P2X_3_ receptors primarily contributed to the activities of P2X currents in muscle DRG neurons (Figure 2a, b). We applied 30 μM α,β‐ATP directly on the muscle afferent DRG neurons to evoke P2X currents, followed by the blockage of P2X_3_ by 10 μM of A‐317491 and a wash‐out recovery. It was found that both P2X transient current (560.15 ± 319.08 pA before A‐317491, *n* = 7, vs. 65.95 ± 16.54 pA immediately after A‐317491, *n* = 7, *P* < 0.05) and sustained current (503.66 ± 307.82 pA before A‐317491, *n* = 10, vs. 79.06 ± 20.44 pA immediately after A‐317491, *n* = 10, *P* < 0.05) were significantly reduced by A‐317491. Those decreased P2X currents were recovered after the wash‐out period: P2X transient current: 65.95 ± 16.54 pA immediately after A‐317491, *n* = 7, vs. 392.46 ± 257.99 pA after wash‐out, *n* = 7, *P* < 0.05; and sustain current: 79.06 ± 20.44 pA immediately after A‐317491, *n* = 10, vs. 466.96 ± 207.23 pA after washing‐out, *n* = 9, *P* < 0.05.

To further verify the P2X current in the muscle afferent DRG neurons is predominantly induced by P2X_3_, P2X_3_ siRNA was applied to specifically knock down the P2X_3_ receptors in the isolated muscle afferent DRG neurons. It was found that, compared with the scramble control, the number and percentage of DRG neurons with P2X currents detected were significantly lower in muscle afferent DRG neurons in the P2X_3_ siRNA group (scramble: 32 in 40 neurons, 80.00% vs. P2X_3_ siRNA: 3 in 31 neurons, 9.68%, *P* < 0.05; Figure 2c).

### 3.3 P2X currents in IB4‐positive muscle DRG neurons

**FIGURE 3:**
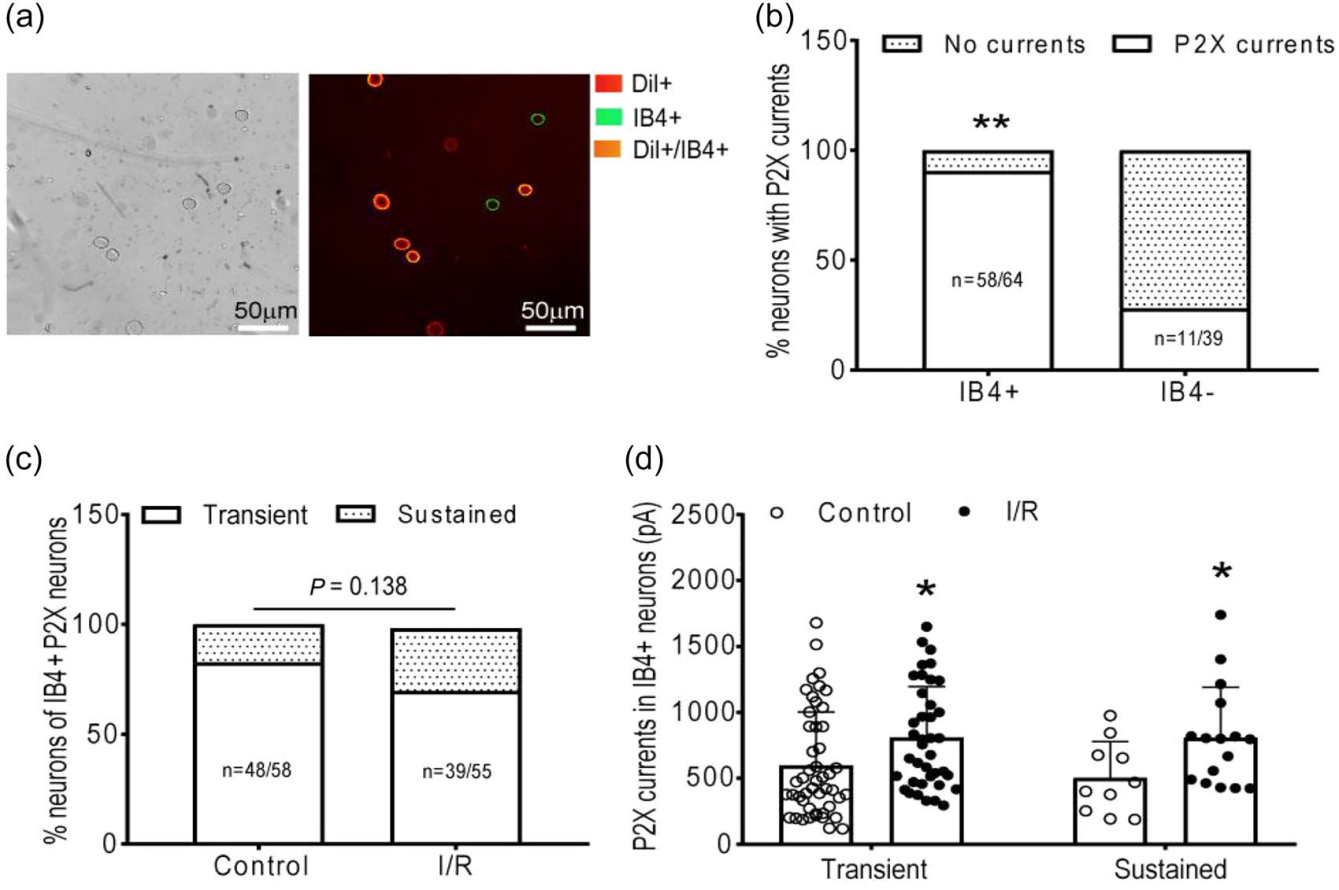
IR increased P2X currents in IB4‐positive muscle DRG neurons. Data presented as means ± SD in (b–d). (a) Co‐localization of DiI (red) identified as hindlimb muscle innervation and IB4‐positive (green) in DRG neurons for the whole cell patch clamp. (b, c) Data were analysed by chi‐square test. The number and percentage of DRG neurons with P2X currents detected were significantly higher in IB4‐positive DRG neurons than in IB4‐negative DRG neurons. **P < 0.01, IB4‐positive neurons versus IB4‐negative neurons. The distribution of P2X current, namely, transient and sustained currents, in muscle DRG neurons was not different between control rats and IR rats (*P* > 0.05). Numbers in parentheses represent the number of neurons with P2X currents/total numbers of the neurons evaluated. (d) The amplitude of P2X currents in muscle DRG neurons was significantly higher in IR rats than in sham rats. *P < 0.05, between IR and sham control.

Figure 3a shows the dual‐labelled DiI‐ and IB4‐positive DRG neuron used for recording whole‐cell patch clamp. It was found that the number and percentage of DRG neurons with P2X currents detected were significantly higher in IB4‐positive DRG neurons than in IB4‐negative DRG neurons (IB4‐positive: 56 in 62 neurons, 90.32% vs. IB4‐negative: 11 in 39 neurons, 28.20%, *P* < 0.01; Figure 3b). Then we examined the P2X current activities in IB4‐positive muscle DRG neurons. Whereas IR did not change the distribution of transient currents and sustained currents in IB4‐positive muscle DRG neurons, the amplitude of P2X currents in the isolated DRG neurons was significantly greater in IR rats than in sham rats (Figure 3c, d): P2X transient currents: 1281.64 ± 776.74 pA in IR rats, *n* = 25, vs. 717.91 ± 556.2 pA in sham rats, *n* = 9, *P* < 0.05; and sustained currents: 466.94 ± 168.68 pA in IR rats, *n* = 11, vs. 271.49 ± 200.90 pA in sham rats, *n* = 8, *P* < 0.05.

### 3.4 P2X‐mediated EPR response

**FIGURE 4:**
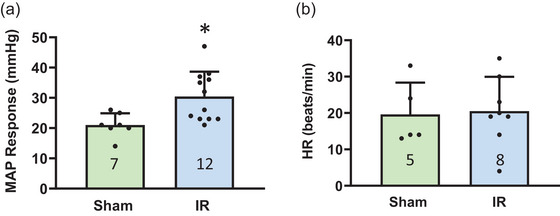
MAP and HR responses to α,β‐meATP in sham and IR. Data presented as means ± SD. Numbers in parentheses represent the sample size of each group. (a) Compared with sham rats, MAP response to α,β‐meATP was exaggerated in IR rats (*P* < 0.05). (b) No significant difference was observed in HR response to α,β‐meATP between sham rats and IR rats (*P* > 0.05).

Compared with sham rats, the mean arterial pressure (MAP) response following the injection of α,β‐meATP (0.125 mM) was significantly amplified in IR rats (α,β‐meATP: 29 ± 8 mmHg in IR rats, *n* = 12 vs. 21 ± 4 mmHg in sham rats, *n* = 7; *P* < 0.05) (Figure 4a). No significant difference was found between the HR response of those groups (21 ± 9 beats/min in IR rats, *n* = 8 vs. 20 ± 9 beats/min in sham rats, *n* = 5; *P* > 0.05; Figure 4b).

### 3.5 P2X_3_ inhibition on MAP response to static muscle contraction

**FIGURE 5:**
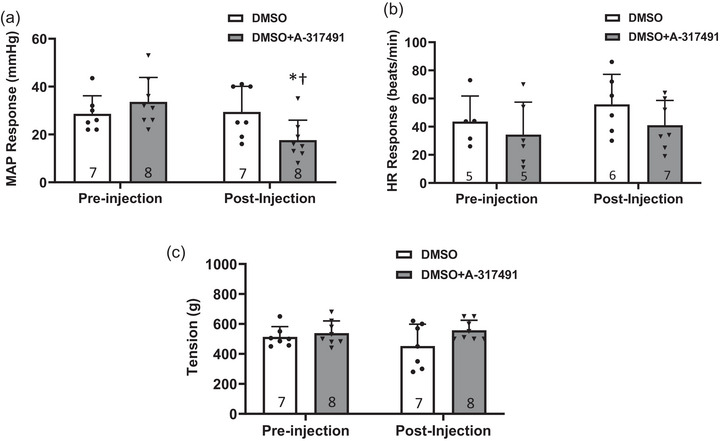
Effect of A‐317491 on MAP response to static muscle contraction in IR. Data presented as means ± SD. White bars indicate IR rats with vehicle control (DMSO) treatment and grey bars indicate IR rats with the vehicle control + A‐317491 (DMSO+A‐317491) treatment. Numbers in parentheses represent the sample size of each group. Pre‐injection: before the injection of DMSO or DMSO+A‐317491. Post‐injection: immediately after completing the injection of DMSO or DMSO+A‐317491. (a) Compared with the pre‐injection control, DMSO+A‐314791 significantly reduced the MAP response to static muscle contraction in IR rats. Compared with DMSO alone, DMSO+A‐317491 attenuates the MAP response to static muscle contraction in IR rats. *Versus IR with DMSO, *P* < 0.05. †Versus pre‐injection control, *P* < 0.05. (b) No significant difference was observed in the HR response among groups (*P* > 0.05). (c) No significant difference was found in development of the muscle tension among groups (*P* > 0.05).

As shown in Figure 5a, before the vehicle control (DMSO) or vehicle control+A‐317491 (DMSO+A‐317491) was injected in the IR rats for the DMSO group or DMSO+A‐317491 group, respectively, no significant difference was found in the static muscle contraction‐induced MAP response between the groups (*P* > 0.05). Immediately after the injection of A‐314791, the MAP response in the DMSO+A‐317491 group was significantly reduced (18 ± 8 mmHg immediately after the injection of A‐314791, *n* = 7, vs. 33 ± 10 mmHg in pre‐injection baseline, *n* = 7, *P* < 0.05). Meanwhile, compared with the IR rats of the DMSO group, the MAP response was significantly lower in the IR rats of the DMSO+A‐317491 group (18 ± 8 mmHg in DMSO+A317491, *n* = 8, vs. 29 ± 11 mmHg in DMSO, *n* = 7, *P* < 0.05). No significant difference was found between MAP response during pre‐drug administration and post‐drug administration in IR rats of the DMSO group (*P* > 0.05). No significant difference was found in the HR response (Figure 5b) and developed muscle tension (Figure 5c) among groups.

### 3.6 P2X_3_ inhibition on MAP response to α,β‐meATP

**FIGURE 6:**
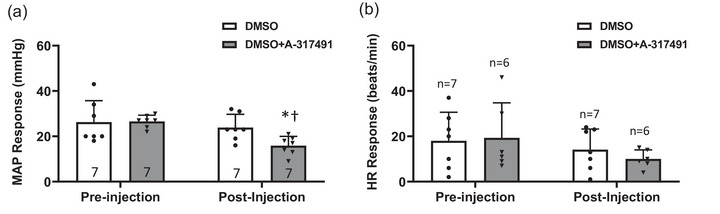
Effect of A‐317491 on MAP response to α,β‐meATP injection in IR. Data presented as means ± SD. White bars indicate IR rats with vehicle control (DMSO) treatment and grey bars indicate IR rats with the vehicle control + A‐317491 (DMSO+A‐317491) treatment. The sample size of each group is represented as numbers in parentheses (a) and as indicated in (b). Pre‐injection: before the injection of DMSO or DMSO+A‐317491. Post‐injection: immediately after completing the injection of DMSO or DMSO+A‐317491. (a) Compared with the pre‐injection control and DMSO injection, A‐314791 significantly reduced the MAP response to α,β‐meATP. *Versus IR group with DMSO, *P* < 0.05. †Versus pre‐injection control, *P* < 0.05. (b) No significant difference was found in the HR response among groups (*P* > 0.05).

As shown in Figure 6a, before the vehicle control (DMSO) or vehicle control+A‐317491 (DMSO+A‐317491) was injected into the IR rats for the DMSO group or DMSO+A‐317491 group, respectively, no significant difference was found in the α,β‐meATP‐induced MAP response between the groups (*P* > 0.05). Immediately after the injection of A‐314791, the MAP response in the DMSO+A317491 group was significantly reduced (16 ± 4 mmHg immediately after the injection of DMSO+A‐314791, *n* = 7, vs. 27 ± 3 mmHg at pre‐injection baseline, *n* = 7, *P* < 0.05). Meanwhile, compared with the IR rats treated by DMSO (DMSO group), the α,β‐meATP‐induced MAP response was also lower in IR rats of the DMSO+A‐317491 group (16 ± 4 mmHg in DMSO+A317491, *n* = 7 vs. 24 ± 6 mmHg in DMSO, *n* = 7, *P* < 0.05). No significant difference was found in the HR response (Figure 6b) among groups.

## 4 DISCUSSION

In this study, we examined the role of P2X_3_ in IB4‐positive DRG neurons in the regulation of amplified EPR response following IR. The key findings are that: (1) compared with sham, IR significantly increased the protein expression of P2X_3_ in L4–6 DRGs (Figure 1); (2) both pharmacological blockade and genetic knockdown of P2X_3_ largely attenuated the P2X currents in muscle DRG neurons (Figure 2), indicating an important contribution of P2X_3_ to developing the P2X current response in muscle DRG neurons; (3) P2X currents were largely detected in IB4‐positive muscle DRG neurons and, compared with sham, IR significantly increased the transient and sustained P2X currents in specific IB4‐positive muscle DRG neurons (Figure 3); and (4) the MAP response to arterial injection of α,β‐meATP stimulating P2X_3_ receptors in the muscle afferent nerves of the hindlimb was significantly increased in IR rats when compared with sham rats, and amplified MAP response to α,β‐meATP and static muscle contraction in IR rats was attenuated by arterial administration of A‐317491 (Figures 4, 5, 6). Overall, our data support the notion that augmented P2X_3_ signalling pathway activity in muscle afferent nerves plays a role in regulating exaggeration of the EPR following the hindlimb IR of PAD.

IR injury is caused by a burst of reactive oxygen species production during reperfusion, which leads to cell damage and inflammation and further exacerbates the underlying ischaemic condition (Tan et al., 2016). PAD patients endure this pathological condition during various situations with this disease, such as (1) the building of collateral blood supply following a main blood flow blockage (Gao et al., 2020; Prior et al., 2004); (2) intermittent claudication: ischaemia and pain occur during walking, followed by reperfusion on stopping walking (Cryer, 1997; Edwards et al., 1994; Hiatt & Brass, 2013; Maxwell & Lip, 1997); and (3) the surgical approach of limb vessel by‐pass or revascularization (Haimovici, 1960; Lau et al., 1991). In one of our previous studies using a hindlimb IR model, the blood flow in the lower extremity of the plantar muscle and gastrocnemius muscle was reduced at 6 h after the femoral artery ligation and gradually restored at 18, 66 and 114 h after the blood flow reperfusion in the femoral artery (Qin & Li, 2022). Meanwhile, the MAP responses to static muscle contraction increased in the above blood reperfusion time courses. This model effectively mimics the haemodynamic changes observed in IR and the exercise‐induced blood pressure response in PAD. This makes it a highly suitable animal model to examine the underlying mechanisms leading to the exaggerated EPR in the IR injury of PAD, to provide a fundmantal base for development of effective interventions to prevent or alleviate PAD‐associated symptoms and complications.

ATP is a primary energy source for muscle contraction and is predominantly present in the intracellular compartment under normal physiological conditions. However, during ischaemic events, the outward movement of ATP across the swollen skeletal muscle cell membrane is enhanced (Boudreault & Grygorczyk, 2002). The measurements in 226 clinical PAD patients suggested that the ATP levels in their plasma were significantly higher than in healthy control participants (Jalkanen et al., 2015). P2X receptors are a class of ionotropic receptors that primarily respond to the presence of ATP, are largely expressed on peripheral thin fibre nerves and central sensory nerves, and play a crucial role in processing various sensory signals (Burnstock, 2017). Among the seven distinct P2X subtypes (P2X_1_–P2X_7_), P2X_2/3_ and P2X_3_ are primarily expressed in DRG neurons (Chen et al., 1995). Of note, McCord et al. (2010) evoked the pressor response in conditions including injection of α,β‐meATP (50 μg/kg) and freely perfused static contraction in a healthy decerebrated animal model. Results of the study indicated that, following the injection of two P_2_X_2/3_ and P_2_X_3_ antagonists, A‐317491 (10 mg/kg) and RO‐3 (10 mg/kg), the pressor response was effectively reduced. Therefore, P_2_X_2/3_ and P_2_X_3_ were identified to be among the primary molecular mediators to regulate the EPR.

Particularly, it is interesting that P2X_2/3_ and P2X_3_ receptors appear in specific groups of IB4‐positive DRG neurons (Cook et al., 1997; Grubb & Evans, 1999; Lewis et al., 1995; Ueno et al., 1999). The growth of IB4‐positive DRG neurons is regulated by glial cell line‐derived neurotrophic factor, while the IB4‐negative DRG neurons are regulated by nerve growth factor (Alvarez et al., 2012). In the present study, we aimed to assess the involvement of the P2X_3_ signalling pathway during EPR regulation following hindlimb IR. Results showed that IR induces increased P2X_3_ expression in the L4–6 afferent DRGs, which is generally consistent with a previous study showing the upregulated P2X_3_ mRNA expression in T1–4 DRGs of a forelimb IR mouse model (Queme et al., 2020). In the patch clamp experiment, we identified enhancement of P2X currents as predominantly detected in IB4‐positive muscle afferent DRG neurons. Identifying the enhanced protein expression of P2X_3_ and its location in muscle afferent DRG serves as a critical step to reveal the potential involvement role of the P2X_3_ pathway in regulating EPR in IR.

To confirm the role of the P2X_3_ pathway in regulating EPR in IR, we further utilized A‐317491 to inhibit P2X_3_ in both whole animal preparations and whole‐cell patch clamp on the isolated muscle DRG neurons. A‐317491 was previously found to have a high affinity with the P2X_3_ and P2X_2/3_ receptors and effectively inhibited calcium influx induced by the receptor mechanism (Jarvis et al., 2002). In a pain behaviour study, administration of A‐317419 significantly reduced pain‐related behaviour in neuropathic and inflammatory conditions (Jarvis et al., 2002; McGaraughty et al., 2003). Similar to the condition in healthy animals, results of the present study showed that A‐317491 significantly attenuated the BP responses to stimulation of muscle afferents by both static muscle contraction and arterial injection of α,β‐meATP in IR rats when compared to the respective pre‐injection baselines. Furthermore, results of in vitro electrophysiology further showed that A‐317491 blocked the P2X currents in isolated muscle DRG neurons. In addition, we utilized the siRNA technique to genetically knock down expression of P2X_3_ in isolated muscle DRG neurons to address concern over the specificity of the pharmacological inhibitors of P2X_3_. Consistent with the results using pharmacological blocking by A‐317491, the siRNA P2X_3_ knockdown largely reduced the number of neurons evoking P2X currents. This indicates that the inhibition of P2X alleviates the exaggerated EPR via reducing P2X_3_‐mediated muscle afferent DRG neuron activity in the IR muscle afferents. Nonetheless, our findings suggest that the P2X_3_ in muscle afferents plays a critical role in regulation of the EPR response in IR although the role of P2X_2/3_ cannot be completely excluded.

In light of the results in the present study, the clinical use of amplified EPR responses in PAD patients could be beneficial by inhibiting or abolishing the enhanced P2X_3_ signalling pathway. Since cardiac output and peripheral resistance are among the chief variables influence the MAP response, it could be speculated that cardiac output and peripheral resistance during exercise were also ameliorated in IR after the inhibition of the P2X_3_ signalling pathway. In translation into the clinical setting, blockage of the P2X_3_ signalling pathway may reduce the burden of the cardiovascular system during exercise. Therefore, potential benefits would be manifested as the reduction of cardiovascular events during daily physical activities (e.g., walking), improved tolerance and adherence to an exercise prescription protocol, etc. To date, a wealth of literatures has indicated the role of aberrant purinergic signalling in hypertension, a known risk factor and one of the chief complications in PAD patients. Notably, in one of the previous studies (Pijacka et al., 2016), researchers were trying to reveal a novel treatment target for hypertension so as to address the drug resistance issue of the current hypertension treatment: they discovered that the expression of P2X_3_ mRNA was significantly increased in petrosal sensory neurons in spontaneous hypertensive rats (SHR). With P2X_3_ antagonism, both in vitro neuron excitability and In vivo blood pressure were reduced in the SHRs. As EPR is also enhanced in many cardiovascular disease patients, the result of the present study also implies potential clinical implications of chronically targeting P2X_3_ signalling in cardiovascular disease with regard to other scenarios where the EPR is exaggerated.

## 5 CONCLUSION

Taken together with the multi‐faceted approaches of In vivo and in vitro, pharmacological agonist/antagonist and genetic knockdown, we conclude that P2X_3_ pathway activity in thin fibre muscle afferent DRG neurons, which is mainly demonstrated to be IB4‐positive, is amplified during the regulation of the EPR response following hindlimb IR. This brings novel insights to the hyperactive EPR response in PAD, and more importantly, to the clinical utilization of the purinergic inhibitors and the future development of potential treatment targets for cardiovascular control in PAD patients.

## AUTHOR CONTRIBUTIONS

Lu Qin designed experiments, performing immunocytochemical and western blot analysis, and drafting the manuscript. Qin Li contributed to data collection and analysis of electrophysiology data and siRNA knockdown data. Jianhua Li designed experiments, oversaw performance of the experiments and data analysis, drafted and revised the manuscript. All authors have read and approved the final version of this manuscript and agree to be accountable for all aspects of the work in ensuring that questions related to the accuracy or integrity of any part of the work are appropriately investigated and resolved. All persons designated as authors qualify for authorship, and all those who qualify for authorship are listed.

## CONFLICT OF INTEREST

The authors declare no conflict of interest.

## Data Availability

Data are available via reasonable request to the corresponding authors.
